# Do Maternal Knowledge and Attitudes towards Childhood Immunizations in Rural Uganda Correlate with Complete Childhood Vaccination?

**DOI:** 10.1371/journal.pone.0150131

**Published:** 2016-02-26

**Authors:** Bryan J. Vonasek, Francis Bajunirwe, Laura E. Jacobson, Leonidas Twesigye, James Dahm, Monica J. Grant, Ajay K. Sethi, James H. Conway

**Affiliations:** 1 Department of Pediatrics, School of Medicine and Public Health, University of Wisconsin-Madison, Madison, Wisconsin, United States of America; 2 Department of Community Health, Mbarara University of Science and Technology, Mbarara, Uganda; 3 Department of Population Health Sciences, School of Medicine and Public Health, University of Wisconsin-Madison, Madison, Wisconsin, United States of America; 4 Department of Sociology, University of Wisconsin-Madison, Madison, Wisconsin, United States of America; Public Health England, UNITED KINGDOM

## Abstract

Improving childhood vaccination coverage and timeliness is a key health policy objective in many developing countries such as Uganda. Of the many factors known to influence uptake of childhood immunizations in under resourced settings, parents’ understanding and perception of childhood immunizations has largely been overlooked. The aims of this study were to survey mothers’ knowledge and attitudes towards childhood immunizations and then determine if these variables correlate with the timely vaccination coverage of their children. From September to December 2013, we conducted a cross-sectional survey of 1,000 parous women in rural Sheema district in southwest Uganda. The survey collected socio-demographic data and knowledge and attitudes towards childhood immunizations. For the women with at least one child between the age of one month and five years who also had a vaccination card available for the child (N = 302), the vaccination status of this child was assessed. 88% of these children received age-appropriate, on-time immunizations. 93.5% of the women were able to state that childhood immunizations protect children from diseases. The women not able to point this out were significantly more likely to have an under-vaccinated child (PR 1.354: 95% CI 1.018–1.802). When asked why vaccination rates may be low in their community, the two most common responses were “fearful of side effects” and “ignorance/disinterest/laziness” (44% each). The factors influencing caregivers’ demand for childhood immunizations vary widely between, and also within, developing countries. Research that elucidates local knowledge and attitudes, like this study, allows for decisions and policy pertaining to vaccination programs to be more effective at improving child vaccination rates.

## Introduction

In 2013 approximately 6.2 million children under the age of five died worldwide, and 3 million of these deaths occurred in Sub-Saharan Africa (SSA) [1]. In 2009, the World Health Organization (WHO) estimated that if global vaccine coverage increased to 90% by 2015, then approximately two million deaths of children under the age of five would be prevented [2]. In the Sub-Saharan African country Uganda, vaccine coverage rates remain well below the WHO goal of 90%, with 82% of children receiving the measles vaccine and 78% completing the three dose series of pentavalent vaccine providing protection against diphtheria, tetanus, pertussis, hepatitis B, and Haemophilis influenza type B (DPT-HB-Hib) in 2013 [3]. One recent study demonstrated that the western region of Uganda, where this study was conducted, has the lowest rate of complete childhood vaccination in the country [4]. Immunizations are a key strategy for reducing the prevalence of infectious diseases, and especially in under-resourced areas, immunizations are a highly cost-effective foundation for developing health systems to invest in [5].

In 2008, the WHO Strategic Advisory Group of Experts on Immunization called for increased information about the factors leading to non-vaccination and under-vaccination of children in order to develop strategies to improve the uptake of childhood immunizations [6]. In rural areas of developing countries, there has been relatively little research into parents’ knowledge and attitudes towards childhood immunizations (KATCI) [7]. Surveying KATCI is an important first step towards understanding the factors that influence vaccine non-acceptance in a particular setting. However, to develop strategies that will improve vaccination rates, the relationship between KATCI and whether they actually have their children adequately vaccinated must be investigated. Community-based sampling, as opposed to, for example, surveying caregivers at a healthcare facility, is particularly important in this context because it ensures broad recruitment inclusive of those most at-risk for under-vaccination [8, 9].

In this study, we aim to first determine basic KATCI by women of childbearing age living in Sheema District, Uganda and to demonstrate how these maternal KATCI correlate with the full, on-time vaccination status of the children of these women.

## Methods

### Study area and population

Data collection took place from September to December 2013 in rural Sheema district in southwest Uganda (population 215,000) located 280 kilometers (km) southwest of Kampala, the capital of Uganda, within access to Mbarara University Science and Technology (MUST; 33 km). Within Sheema district, the study was conducted in 10 villages from two parishes. In the parish of Kiziba of Kagango subcounty, the villages sampled included: Bisharara, Butagasi, Kagorogoro, Mbagwa, and Ntungamo. In Rweibare parish of Kyangyenyi subcounty, the villages sampled included Buhihi, Katooma, Kyangundu, Rweibare II, and Rweibare IV. These villages were selected for two main reasons. First, they are in close proximity to MUST, the overseeing academic institution. Second, these villages are densely populated areas located farther from the main road than other sub-counties, and residents may face greater challenges accessing health services. Kyangyenyi and Kagango sub counties have a combined population of 70,500 people residing in 13,121 households, with an estimated 2,115 births per year based on annual population growth of 3%. In this area, the large majority of vaccination services are provided by governmental health facilities. Within Sheema District, there are a total of 27 government health facilities, 8 NGO health facilities, and no private health facilities [10].

### Study sample and design

Within the purposively selected villages, 1000 women were surveyed for the cross-sectional study. A needed sample size of 969, which was rounded to 1000, was calculated for two-sided testing of differences in proportions as little as 10% for relatively common factors of interest (overall prevalence = 50%), α = 0.05, and 1-β = 0.80 with a 15% level of non-participation. To allow for village- and region-level analyses with modest statistical power, we set out to recruit a random sample of 100 eligible women from 10 different villages. However, once in the field, we found fewer than 100 eligible women in some villages; thus, women from adjacent villages were approached for the study. No more than one woman per household was recruited.

For each of the households approached, it was first determined which household members were eligible for participation. Eligible participants were female, age 15 to 50 years, verbally confirmed that they had slept at the house the night before, and were willing to consent to the study procedures. If only one person met the eligibility criteria, that individual was asked to participate in the study. If more than one person from the household met the eligibility criteria, then one of them was randomly selected and asked to participate in the study. Of those enrolled in the study, 97.4% were surveyed during the initial visit to the household. Others were not available during the initial visit so interviewers had to return to the household a second (1.8%) or third (0.8%) time to conduct the interview with the selected individual. When recruitment was unsuccessful, either due to resident refusal or three unsuccessful attempts to meet with the selected individual, the next household along the road was approached.

The 1000 women enrolled in the study were interviewed using a standardized questionnaire that collected socio-demographic data and their KATCI. To gauge knowledge, women were surveyed on their understanding of the purpose of vaccinations and the process and timing of immunizing children. To analyze knowledge of vaccine preventable diseases (VPDs), women were asked whether certain diseases are vaccine preventable. Three categories of diseases were asked about:

1. Diseases preventable by WHO recommended vaccines in Uganda at the time of this study (WHO VPDs): polio, hepatitis, whooping cough, diphtheria, tetanus, measles, and meningitis.
2. Other “correct” vaccine preventable diseases (non-WHO VPDs): pneumonia, cancer, diarrhea, and yellow fever.
3. Diseases not preventable with vaccines: HIV and malaria.

To quantify attitudes towards childhood immunizations, the questionnaire covered the importance of immunizations and reasons for low vaccination coverage in these communities.

**Fig 1:**
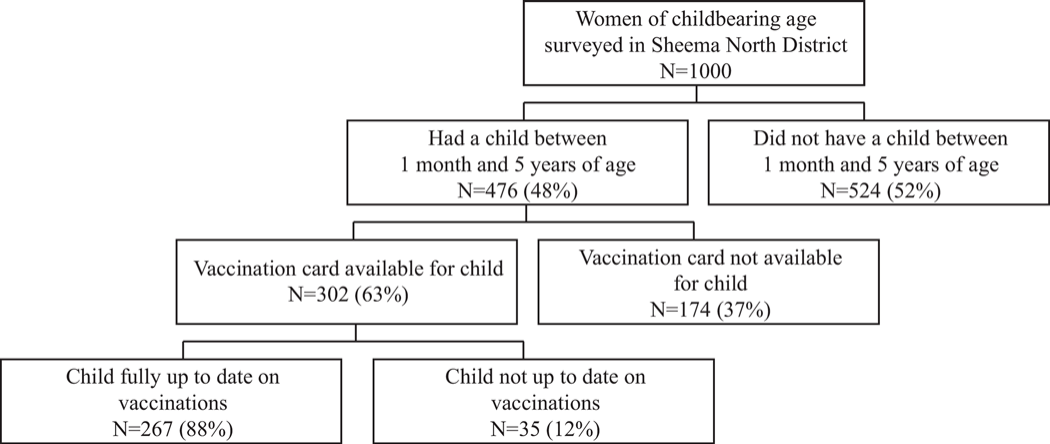
Derivation of the sample of children and mothers.

Vaccination data and date of birth was obtained for children between the ages of one month and five years. The Uganda Ministry of Health provides health cards to caregivers of every child. These cards contain records of children’s birth history, vaccination history, and other pertinent past medical history. Vaccination data were only obtained from vaccination cards sighted during the household visit (Fig 1). When participants included in the study had more than one child within the age range and with a vaccination card available, only vaccination data from the single child closest to one year of age was collected.

### Data collection, management, and analysis

The questionnaires were prepared in English and translated into the local language Runyankole. A reviewer fluent in both languages verified and helped revise the translation. With a group 2% of the sample size and similar demographic characteristics as the study group, the questionnaire was pre-tested and appropriate revisions were made afterwards. Face-to-face interviews were conducted in Runyankole with data collection on paper forms. Ten current students and recent graduates from Mbarara University of Science and Technology fluent in English and Runyankole participated in the data collection. They were trained on the purpose of the study, use of the questionnaire, ethical obligations as data collectors, and practicalities of conducting field surveys. On a daily basis, the field coordinator (LT) checked the completeness and consistency of the collected data.

Data from the paper questionnaires was entered once into a computer using EpiData software. Data were then exported to Stata (version MP11.0, Stata Corporation) for cleaning and analysis. Frequencies, percentages, means, and standard deviations of the women’s socio-demographic characteristics and KATCI were produced. At a descriptive level, these variables were compared between the entire study sample and those with a child between one month and five years of age with a vaccination card available for this child at the time of the interview (U5+Card). This was done using Pearson’s chi-square statistic for categorical variables and Kruskal-Wallis equality-of-proportions rank test for continuous variables.

For women in the U5+Card group, the dependent variable was dichotomized into fully vaccinated and not fully vaccinated as described below. To identify the factors associated with the immunization status of children, bivariate logistic regressions and generalized linear models were used to determine prevalence ratios (with 95% confidence intervals) for continuous and categorical variables, respectively. Statistical significance was considered to be *p*-value < 0.05.

There is growing awareness of the importance of timeliness of childhood immunizations when assessing actual protection from VPDs [11–14]. We therefore made timeliness of vaccination a requirement for full vaccination status with the following cutoffs: Polio “at birth” by 30 days after birth, BCG + second dose of polio + first dose of DPT-HB-Hib by 2 months, third dose of polio + second dose of DPT-HB-Hib by 4 months, fourth dose of polio + third dose of DPT-HB-Hib by 6 months, and measles vaccine by 12 months. Infants less than one month old were not included in the analysis because of the leeway given with the first polio dose and BCG, which are “scheduled” to be given at birth.

The majority of vaccinations were dated on the available cards. There were nine individuals that had one or two vaccinations (out of nine total vaccinations data were collected on) recorded as “given” on the card but the dates at which these vaccines were given were not registered. For these particular vaccines it was assumed that they were given at the appropriate time because all the other recorded vaccines given to each of these individuals were given on time.

A socioeconomic wealth index was developed through a principal component analysis (PCA) based on household characteristics such as electricity and number of rooms and ownership of assets such as mobile phone, motorcycle, and livestock. There are many difficulties in creating wealth indices in low-income countries, and a variety of methods, each with their own limitations, are available. We chose to use PCA because of its versatility with both discrete and continuous variables [15]. Households were ranked by socioeconomic wealth index and grouped into quartiles.

### Ethical considerations

The study was approved by the Uganda National Council for Science and Technology (UNCST), Mbarara University of Science and Technology Internal Review Board (10/04-13), and the University of Wisconsin-Madison Internal Review Board (2013–0665). Oral research permissions were solicited and given by the appropriate local government and traditional leaders. The interviewers were trained to properly obtain informed consent and did so will all study participants. The study was explained verbally in the local language to each participant. Written consent was obtained before the research assistant continued with the interview. If the age of the participant was between 15 and 17 years, a guardian was required to provide assent and written consent on behalf of the individual. We excluded participants who were unwilling to answer questions about pregnancy or related complications, who were unwilling to provide written consent, or in cases where they were under 18, if no one was available to provide assent and written consent on their behalf.

## Results

### Sample characteristics

Of the total 1000 women of childbearing age interviewed, 302 had a child between one month and five years of age and a vaccination card for this child at the time of the interview (U5+Card). This group of mother-child pairs was then analyzed for factors associated with full vaccination status. Compared to the entire sample, the women in the U5+Card group had lower representation in the wealthiest quartile, were generally younger, were more likely to be in a union, and were more likely to have slept under a bed net the previous night. Other characteristics of sampled women, as well as the age of children eligible for study inclusion based on age, are shown in Table 1.

**Table 1:** Socio-demographic characteristics of all women in the study sample and those with a child between one month and 5 years old with a vaccination card (U5+Card).

	U5+Card	Total
Characteristic	N (%)	N (%)
Wealth Index (quartile)		
Poorest (reference)	80 (26.5)	250 (25.0)
Second	80 (26.5)	250 (25.0)
Third	79 (26.2)	250 (25.0)
Wealthiest	63 (20.9)	250 (25.0)
Parish		
Kiziba (reference)	133 (44.0)	504 (50.4)
Rweirbare	169 (56.0)	496 (49.6)
Age of mother (years)		
15–24	98 (32.5)	285 (28.5)
25–30	117 (38.7)	259 (25.9)
31–38	58 (19.2)	227 (22.7)
39–50	29 (9.6)	229 (22.9)
Age of child		
0–6 months	30 (9.9)	35 (3.5)
6–12 months	52 (17.2)	63 (6.3)
1–5 years	220 (72.9)	378 (37.8)
No child in age range	0 (0.0)	524 (52.4)
Mean (SD) number of household members	4.58 (2.33)	4.59 (2.42)
Marital status		
Not in a union (reference)	18 (6.0)	208 (20.8)
In a union	284 (94.0)	792 (79.2)
Currently pregnant		
No (reference)	278 (92.1)	903 (90.3)
Yes	24 (7.9)	97 (9.7)
Wants more children		
No (reference)	121 (40.1)	482 (48.2)
Yes	181 (59.9)	518 (51.8)
Slept under a bed net previous night		
No (reference)	144 (47.7)	558 (55.8)
Yes	158 (52.3)	442 (44.2)

### Knowledge of childhood immunizations

Table 2 shows various aspects of knowledge of childhood immunizations by all women surveyed and women in the U5+Card group. Compared to the larger group, those in the U5+Card group demonstrated similar knowledge other than being more likely to understand how often an infant needs to be vaccinated (71.2% versus 63.9%, P = 0.0194). None of these other measures of vaccine knowledge were significantly different between the U5+Card subgroup and total sample. The majority of women were able to state that childhood immunizations protect children from diseases (93.5%). Many also thought that they strengthen or improve children’s health (32.8) or promote children’s growth (26.3%). The most common VPDs identified were polio (81.3%) and measles (77.5%). 8.6% of women thought that malaria is vaccine preventable. A majority of women correctly stated how often an infant needs to be vaccinated (63.9%) or the nearest facility for vaccinations (88.9%).

**Table 2:** Knowledge of childhood immunizations and perceived probability that next child will receive required immunizations by all women in the study sample and those with a child between one month and 5 years age with a vaccination card (U5+Card).

	U5+Card	Total
Characteristic	N (%)	N (%)
Stated reasons to immunize children		
Protect children from disease	278 (92.1)	935 (93.5)
Promote child’s growth	92 (30.5)	328 (32.8)
Strengthen/improve child’s health	77 (25.5)	263 (26.3)
Treat/cure disease	21 (7.0)	57 (5.7)
Don't know	6 (2.0)	17 (1.7)
Diseases that mother reports immunization can protect against*		
Polio	247 (81.8)	813 (81.3)
Measles	217 (71.9)	775 (77.5)
Tetanus	141 (46.7)	459 (45.9)
Whooping cough	136 (45.0)	455 (45.5)
Tuberculosis	120 (39.7)	402 (40.2)
Diphtheria	62 (20.5)	214 (21.4)
Hepatitis	25 (8.3)	73 (7.3)
Meningitis	3 (1.0)	15 (1.5)
Diarrhea	39 (12.9)	114 (11.4)
Pneumonia	14 (4.6)	44 (4.4)
Yellow Fever	5 (1.7)	14 (1.4)
Cancer	3 (0.3)	1 (0.1)
Malaria	30 (9.9)	86 (8.6)
HIV	5 (1.7)	17 (1.7)
Mentioned others	9 (3.0)	28 (2.8)
Don't know	3 (1.0)	11 (1.1)
Understands how often an infant needs to be vaccinated	215 (71.2)	639 (63.9)
Knows the location of the nearest facility for vaccinations	278 (92.1)	888 (88.9)
Mother’s perceived probability next child will be fully immunized [mean (SD)]**	0.74 (0.33)	0.75 (0.31)

*Respondents could report more than one response

**Among 209 women with a U5+ Card and 594 total women who expected to have another child in the next five years.

**Fig 2:**
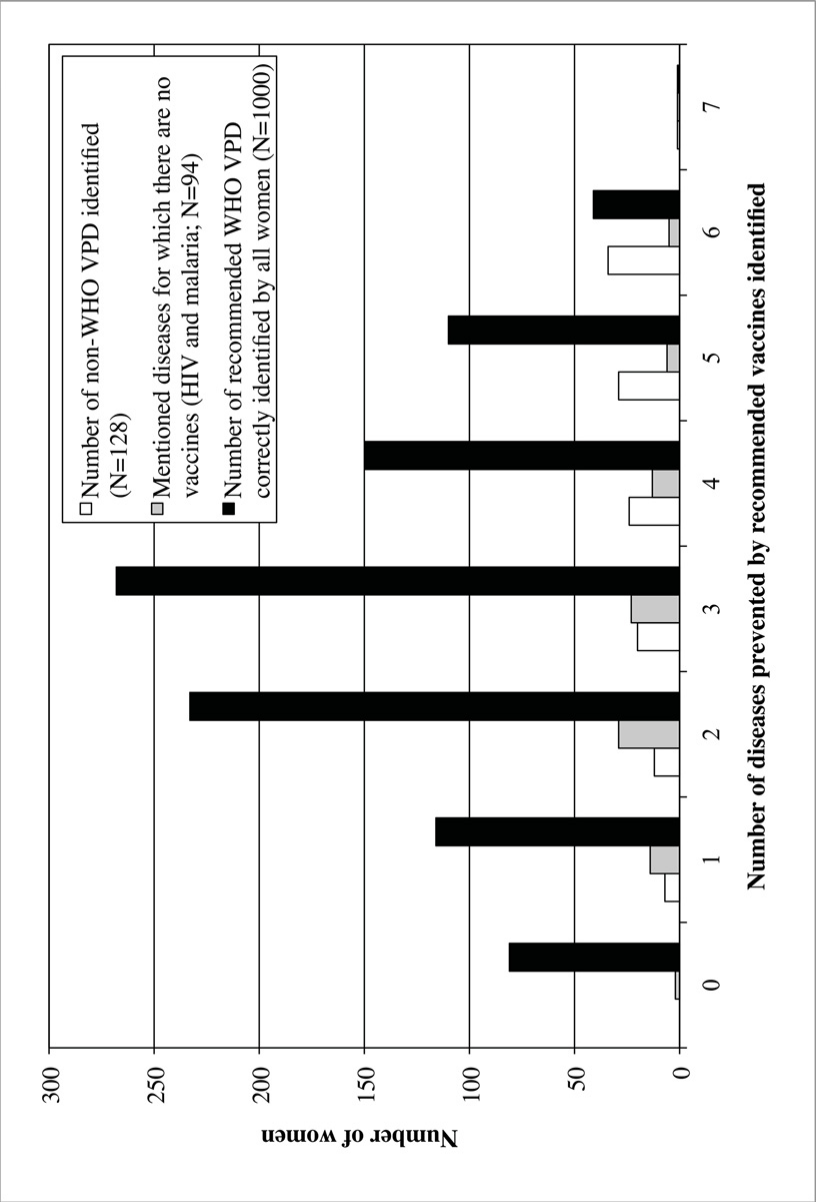
Number of diseases identified correctly and incorrectly as vaccine preventable by women surveyed (N = 1000).

Fig 2 illustrates the distribution of women identifying certain diseases as vaccine preventable. The women correctly identified a mean of 2.98 (SD = 1.39) out of seven recieveds. Women reporting more WHO VPDs were more likely to mention non-WHO VPDs (r = 0.804). There was no correlation between mentioning malaria and/or HIV as vaccine preventable and number WHO VPDs identified.

### Attitudes towards childhood immunizations

Women endorsing that they wanted to have another child within the next five years were asked to state the probability that child would be fully immunized. As shown in Table 2, the mean probability reported was 0.75 (SD = 0.31). When asked whether it is “very,” “somewhat,” or “not important” for infants to receive vaccinations, 95.7% stated that it is “very important,” 3.9% stated “somewhat important,” and 0.3% stated “not important.” Table 3 shows coded free text of the women’s responses to the question “Why do you think some mothers in your community do not have their children vaccinated at proper times?” The two most common response categories, “fearful of side effects” and “ignorance/disinterest/laziness,” were both over twice as prevalent (44% each) as the next most prevalent response. Reasons related to vaccine supply, “vaccine shortages” and “crowds or long waits,” were only mentioned by 10% of respondents. Responses of women in the U5+Card group were very similar to the distribution of responses for the entire sample.

**Table 3:** Reasons why parents in their community may not have their children fully vaccinated as reported by all women surveyed (N = 1000) and only those with a child between the ages of one month and five years with a vaccination card (U5+Card, N = 302).

	U5+Card	Total
Reason reported by women	N (%)	N (%)
Travel or financial problems	54 (18%)	162 (18%)
Being fearful of side effects	138 (46%)	441 (44%)
Vaccine shortages	9 (3%)	27 (3%)
Ignorance, disinterest, or laziness	128 (42%)	441 (44%)
Discouragement from husband or family	31 (10%)	112 (11%)
Crowds or long waits	23 (8%)	66 (7%)
Lack of time or being too busy	15 (5%)	50 (5%)
Disrespectful healthcare staff	8 (3%)	36 (4%)
Don’t know	28 (9%)	106 (11%)

### Factors associated with full vaccination status

Of the 302 children whose vaccination cards were analyzed for this study, 267 (88%) were vaccinated in a timely manner with all vaccines they were eligible for based upon their age. The 35 under-vaccinated children most frequently missed the last polio dose (48%) or measles (43%), as shown in Table 4.

**Table 4:** Frequency of specific vaccinations missed by children between the ages of one month and five years that had vaccination history documented but were not fully up to date.

Vaccination	Frequency	Number Eligible	Percent of Children Missed
BCG	9	35	26%
Polio 0	4	35	11%
Polio 1	4	33	12%
Polio 2	4	31	13%
Polio 3	14	29	48%
DPT 1	5	33	15%
DPT 2	4	31	13%
DPT 3	9	29	31%
Measles	10	23	43%

Table 5 explores factors associated with full vaccination status. Women that were able to correctly state that childhood immunizations protect children from diseases were more likely to have their child fully immunized compared to women that were not able to state this benefit of immunizations (PR = 1.354, CI: 1.018, 1.802). Other aspects of vaccine knowledge, including knowledge of specific VPDs and understanding how often and where to get a child vaccinated, were not significantly associated with full vaccination status. The two measures of attitudes towards childhood immunizations, mother’s perceived probability that next child will be fully immunized and the various categories of reasons stated for low vaccination rates in the community, also were not significantly associated with full vaccination status of the child.

**Table 5:** Bivariate analysis of factors associated with full vaccination among children between one month and 5 years old with vaccination cards.

Characteristic	N	PR	95% CI
Wealth Index (quartile)			
Poorest (reference)	80	1.0	—
Second	80	1.075^#^	0.952, 1.213
Third	79	1.058	0.934, 1.199
Wealthiest	63	1.099^#^	0.974, 1.240
Parish			
Kiziba (reference)	133	1.0	—
Rweirbare	169	1.073^#^	0.985, 1.168
Age of mother (years)			
15–24	98	1.0	—
25–30	117	1.009	0.753, 1.354
31–38	58	0.960	0.665, 1.374
39–50	29	0.883	0.532, 1.411
Age of child			
0–6 months	30	1.0	—
6–12 months	52	1.105	0.902, 1.356
1–5 years	220	1.119	0.931, 1.346
Number of household members	302	0.951	0.822, 1.100
Marital status			
Not in a union (reference)	18	1.0	—
In a union	284	0.932	0.571, 1.626
Number of live births	302	0.981^#^	0.960, 1.002
Currently pregnant			
No (reference)	278	1.0	—
Yes	124	1.092	0.679, 1.677
Wants more children			
No (reference)	121	1.0	—
Yes	181	1.064^#^	0.975, 1.162
Slept under a bed net previous night			
No (reference)	144	1.0	—
Yes	158	0.946	0.739, 1.212
Has mobile phone access			
No (reference)	90	1.0	—
Yes	212	1.087^#^	0.981, 1.204
Mother’s perceived probability that next child will be fully immunized^a^	209	1.012^#^	0.996, 1.028
Travel distance for vaccinations (kilometers)			
All subjects	302	0.975*	0.954, 0.996
Rely on walking	242	0.968*	0.940, 0.996
Rely on public transport	48	0.995	0.946, 1.048
Transportation to vaccination facility			
Rely on public transport (reference)	105	1.0	—
Rely on walking	469	1.103^#^	0.972, 1.252
Stated importance of vaccinating children			
Very important (reference)	292	1.0	—
Somewhat or not important	10	1.136	0.538, 2.124
Beliefs as to why others do not vaccinate their children			
Travel or financial problems			
No (reference)	248	1.0	—
Yes	54	0.981	0.700, 1.350
Being fearful of side effects			
No (reference)	164	1.0	—
Yes	138	0.956	0.744, 1.225
Vaccine shortages			
No (reference)	293	1.0	—
Yes	9	0.748	0.272, 1.651
Ignorance, disinterest, or laziness			
No (reference)	174	1.0	—
Yes	128	1.077	0.838, 1.380
Discouragement from husband or family			
No (reference)	271	1.0	—
Yes	31	0.983	0.635, 1.467
Crowds or long waits			
No (reference)	279	1.0	—
Yes	23	0.982	0.590, 1.550
Lack of time or being too busy			
No (reference)	287	1.0	—
Yes	15	0.979	0.514, 1.705
Disrespectful healthcare staff			
No	294	1.0	—
Yes	8	1.135	0.485, 2.270
Understands how often an infant needs to be vaccinated			
No (reference)	87	1.0	—
Yes	215	0.998	0.762, 1.319
Knows the location of the nearest facility for vaccinations			
No (reference)	24	1.0	—
Yes	278	1.011	0.647, 1.663
Knowledge score regarding specific vaccines	302	1.014^#^	0.994, 1.034
Stated reasons to immunize children			
Protect children from disease			
No (reference)	24	1.0	—
Yes	278	1.354*	1.018, 1.802
Promote child’s growth			
No (reference)	77	1.0	—
Yes	210	1.018	0.763, 1.346
Strengthen/improve child’s health			
No (reference)	210	1.0	—
Yes	92	1.048	0.799, 1.364
Treat/cure disease			
No (reference)	281	1.0	—
Yes	21	1.025	0.607, 1.635

PR = prevalence ratio

^#^p<0.25

*p<0.05

^a^Mothers were only asked this question if they expected to have another child in the next five years

The prevalence of children fully vaccinated decreased as reported distance traveled to site of vaccination increased (PR = 0.975, CI: 0.954, 0.996). When stratified by type of travel, this association increased in the group that reported that they walk to vaccination sites (PR = 0.968, CI: 0.940, 0.996). There was no association in the groups that reported that they use public transport, bicycle (n = 11), or private vehicle (n = 1). Socio-demographic covariates including wealth of the household, age of the mother or child, number of people in the household, maternal marital status, maternal desire for more children, and maternal parity were not associated with full vaccination status.

## Discussion

This study describes knowledge and attitudes towards childhood immunizations by parous women living in rural western Uganda. In addition, the study describes how these attitudes and knowledge associate with the vaccination status of the children of the women surveyed.

When used to predict factors influencing caregivers’ demand for childhood immunizations, the health belief model predicts that important factors include perception of a child’s susceptibility to VPDs, perception of severity of disease caused by VPDs, impression that immunizations are effective and beneficial, and perception of adequate access to vaccinations [8]. An understanding of the degree to which vaccines are acceptable in communities and the reasons behind any hesitancy from parents to vaccinate their children is important for the success of immunization programs [16]. Studies in SSA analyzing parental characteristics associated with the vaccination status of their respective children have largely focused on other socio-demographic factors. Variables such as parental education [4, 17–23], mothers’ age [18, 19], household income or wealth [4, 11, 17, 19, 22, 24], family size or mothers’ parity [4, 17–19], religion of caregivers [25, 26], and location of labor and delivery [23] are well documented as correlates with the vaccination status of a child. We are only aware of four published studies conducted in SSA in the past 15 years that assessed KATCI [18, 27–29]. Only one of these studies analyzed how maternal attitudes towards immunizations correlate with the vaccination status of a child by undergoing a limited bivariate analysis of mother’s negative attitudes towards local healthcare facilities providing vaccinations and child’s measles vaccination status [18].

In this study, the under-vaccinated group was significantly less likely to know that the main purpose of immunizations is protection from diseases. This is complemented by respondents frequently attributing poor vaccination rates in their community to factors in the category of “ignorance/disinterest/laziness.” VPDs are typically an unseen threat, and the utility of vaccinating children isn’t immediately apparent. When caregivers have basic insight into the larger benefits of vaccinating their children, they may place higher priority on this task amidst multiple other competing interests for their time [8].

Women in this study were asked why parents in their community may not have their children vaccinated, and the two most common responses were “fearful of side effects” and “ignorance/disinterest/laziness.” Two other studies have analyzed caregivers’ perceived reasons for under-vaccination by directly asking the caregivers of under-vaccinated children. Mohamud et al. reported that a population of caregivers with under-vaccinated children in Jigjiga District, Ethiopia most commonly stated “busyness due to work load” as the main reason for not fully vaccinating their respective children [23]. Oria et al. reported that a rural population in western Kenya and an informal settlement in Nairobi most commonly stated “child was sick during vaccination period” and “parent was too busy,” respectively, as the main reason for not vaccinating their children for influenza [30]. Responses similar to “fearful of side effects” and “ignorance/disinterest/laziness” were much less common in both of these surveys. These discrepancies may reflect true differences in barriers to vaccinating children between these different communities. However, the differences may also be attributable to the source of information; this study asked mother’s to generalize about other caregivers in their community while these other two studies asked the caregivers to provide reasons for their own lack of action.

A deeper understanding of the specific VPDs was not associated with the vaccination status of children. These findings are consistent with the majority of evidence from low-income countries, which generally shows that in-depth understanding of vaccinations by caregivers is not an important factor for high immunization coverage [31]. Aspects of caregivers’ KATCI that are thought to correlate with vaccination status of children in low-income countries include positive attitudes towards immunizations and practical knowledge of immunizations such as when to go, how often, and where to go to have a child immunized [31]. We found positive attitudes and practical knowledge to be widespread, but within the constraints of our research methodology, these were not correlates with vaccination status. It is noteworthy that Muhwezi et al. demonstrated in Uganda that some aspects of specific knowledge of cervical cancer and the human papilloma virus (HPV) vaccine correlate with caregivers’ willingness to allow their male children to receive HPV vaccines. Caregivers aware of cervical cancer, aware of a vaccine available to prevent cervical cancer, and aware that HPV causes genital warts were significantly more likely to be willing to have their male children receive the HPV vaccines [32].

Although this group of women most commonly identified polio and measles as VPDs, the 35 under-vaccinated children most commonly missed the last dose of the polio and the measles vaccine. This provides further evidence that knowledge of specific vaccines is poorly associated with their uptake. Administration of BCG and polio at birth is required for any registered maternity health facility in Uganda. Assuming a mother delivers at one of these maternity health facilities, it is possible that factors such as demand and access are relatively less important for receiving these two vaccines. Along these lines, under-vaccinated children in this study were least likely to miss polio at birth, but surprisingly, twice as many did not receive BCG on time.

Many studies in low-income countries have also demonstrated that, particularly in rural areas, long travel distance to vaccination points can be a barrier to immunization uptake [31]. The mothers of children in the under-vaccinated group of this rural population had significantly longer perceived travel distance to the vaccination facility, especially if they walk rather than rely on other modes of transportation. However, less than one-fifth of all respondents reported travel or financial problems as a reason for poor vaccination rates in their community.

In our sample, 88% of children had full vaccination status. Recent estimates from the Uganda Ministry of Health show significantly lower coverage for individual vaccines in Sheema District: 70.3% for BCG, 59.2% for measles, and 66.8% for the final dose of polio [10]. To a lesser extent, the national estimates for individual vaccines in Uganda reported by the WHO and listed in the Background are also lower [3]. These discrepancies are surprising given that we used a more stringent definition of full vaccination status by also requiring timeliness of vaccination. Limiting our analysis only to mother-child pairs with vaccination cards available was the most likely reason for a markedly higher rate of full vaccination status in the population we sampled.

Rwashana et al. developed a model of factors affecting demand for immunization in Uganda using a dynamic synthesis methodology, which combines system dynamics and case study research methods [33]. Their model identifies three main factors influencing demand for immunization in Uganda: level of immunization awareness, mothers’ availability, and level of trust in the health system. As described above, we found level of immunization awareness to correlate with immunization uptake. Many of the reasons reported by these women for low vaccination rates in their communities do coincide with the later two main factors in this model. Future efforts are needed to elucidate the full impact of disinterest or lack of engagement in the vaccination process and concerns about the side effects of vaccinations.

Access to mobile phones for mothers was not significantly different between those with fully and under-vaccinated children. Mobile phone access has been increasing dramatically in rural areas of developing countries such as this over the past decade, and mobile phone-based interventions for improving vaccination coverage in populations at risk for under-vaccination are quickly becoming more feasible and efficacious [34–36]. These interventions may be especially useful in this population where the under-vaccinated group has relatively good mobile phone access.

Knowledge and attitudes towards childhood immunizations vary greatly in different settings [7, 37]. This study was conducted in rural communities populated mostly by subsistence farmers in western Uganda. Although there is much ethnic and socioeconomic diversity in Uganda, it is estimated that 85% of the country’s population lives in rural areas. The results of this study may have implications for vaccination programs in similar settings. However, considering the dearth of information available and high variability in populations that have been studied, we believe basic operational research that elucidates local knowledge and attitudes should be scaled up and considered essential to every vaccination campaign or program. This will help identify root causes of poor demand for vaccinations and allow for effective decisions and policy tailored to fit the needs of communities. This study provides a framework for how this type of operational research can be conducted in developing parts of the world.

Our findings should be interpreted with several limitations of the study in mind. The effect of confounding cannot be underestimated given that we relied on bivariate analysis to determine factors associated with full vaccination coverage. Mother-child pairs were only included in the bivariate analysis if a vaccination card was available at the time of the interview. With no vaccination card available, 174 mother-child pairs eligible based on all other criteria were not included. This exacerbated the issue of small sample size and inadequate power. A study involving mothers in rural South Africa demonstrated that maternal recall of child vaccination dates has high sensitivity when compared to information extracted from vaccination cards [38]. We did attempt to solicit vaccination dates by maternal recall in the group without vaccination cards available. These data were deemed unreliable due to concern for recall bias and because many of the mothers were not able to recall exact dates of vaccinations.

Our survey did not collect information about women’s educational background and place of delivery for mother-child pairs. Both of these variables have been shown to be associated with the vaccination status of children in similar settings [19, 20]. Not accounting for them while analyzing our primary input variables of interest may be a source of bias. Next, although we demonstrated that the prevalence of fully vaccinated children decreases as travel distance to the vaccination site increases, it is important to recognize that we had each of these participants estimate this distance. We didn’t evaluate the accuracy of these estimations, and the amount of bias with these estimations is uncertain. Finally, we only assessed the KATCI of mothers, who certainly have a large influence on the net demand of a particular child’s vaccination. However, a comprehensive analysis of the connection between the vaccination status of a child and the mindset of those caring for the child also must take into consideration KATCI of fathers and other immediate caregivers.

## Conclusions

Most studies analyzing factors influencing caregivers demand for childhood immunizations in rural, resource-limited settings do not focus on caregivers’ KATCI. Our analysis shows that in this rural setting of western Uganda, mothers with a basic understanding of the importance of childhood immunizations were more likely to have timely, full vaccination of their children. Many of these women suggested that poor vaccination rates in their community are due to caregivers’ fear of side effects and disinterest or ignorance towards vaccinations. Prospective, larger scale analyses are needed to delineate the community-specific influence caregivers’ KATCI has on children’s vaccination status. This will allow for the development of more effective interventions and policy to improve vaccination coverage in developing countries.
